# Minimising bias in an un-masked, pragmatic rct comparing two treatment pathways for glaucoma by the use of decision support software - the light trial experience

**DOI:** 10.1186/1745-6215-14-S1-P131

**Published:** 2013-11-29

**Authors:** Gus Gazzard, Haogang Zhu, Amanda Lewis, Neil Nathwani

**Affiliations:** 1 NIHR Biomedical Research Centre at Moorfields Eye Hospital NHS Foundation Trust and UCL Institute of Ophthalmology, London, UK; 2 City University, London, UK

## Introduction

Pragmatic trials of treatment pathways can require patient awareness of treatment allocation in order to better represent clinical reality, for example when concordance with a treatment has an important effect on outcome. Conversely, masking of treating clinicians to allocation group can be impossible when full clinical assessment requires knowledge of the current treatments, resources do not permit separate teams for treatment and assessment or when such duplication of clinician contact might affect an outcome such as patient experience.

The LiGHT trial is a 718 subject multi-centre 6-year NIHR-funded study of two treatment pathways for glaucoma with outcome measures of health related quality of life and cost effectiveness. We aimed to minimise variation in aspects of clinical behaviour that might introduce bias by affecting either of these outcomes.

## Methods

Custom-written decision support software permits real-time decision-making using analysis of multiple clinical measures made by masked observers: optic disc analysis, visual field assessment and intra-ocular pressure measurements. Treatment targets are objectively defined according to disease severity criteria. Target attainment accounts for known measurement uncertainties.

## Results

A treatment algorithm based on robust, published criteria determines clinical decisions that influence cost or patient experience (treatment escalation, follow-up interval and intensity of testing).

## Conclusion

Where objective criteria for clinical decisions exist, it is possible to balance the competing demands of reducing bias with protocol-driven escalation criteria with a pragmatic ‘real-world’ approach to therapeutic choices permitting variation in clinical practice, in a manner acceptable to clinicians recruiting into an unmasked pragmatic trial.

